# Prokaryotic communities in the historic silver mine Reiche Zeche

**DOI:** 10.1007/s00792-021-01249-6

**Published:** 2021-12-08

**Authors:** Götz Haferburg, Tobias Krichler, Sabrina Hedrich

**Affiliations:** 1 Institute of Bioscience, Research Group Biohydrometallurgy and Microbiology, TU Bergakademie Freiberg, Leipziger Strasse 29, 09599 Freiberg, Germany; 2 Institute for Mining and Special Civil Engineering, TU Bergakademie Freiberg, Fuchsmuehlenweg 9, 09599 Freiberg, Germany

**Keywords:** Biofilms, Acidophiles, Candidate phyla radiation, Next-generation sequencing, Historic mine

## Abstract

The research and education mine “Reiche Zeche” in Freiberg (Saxony, Germany) represents one of the most famous mining facilities reminiscent to the century-long history of silver production in the Ore Mountains. The mine was set up at the end of the fourteenth century and became part of the “Bergakademie Freiberg” in 1919. Galena, pyrite, sphalerite, arsenopyrite, and chalcopyrite are the most common minerals found in the mine. As acid mine drainage is generated from the dissolution of sulfidic ores, the microbial habitats within the adits and galleries are characterized by low pH and high concentrations of metal(loid)s. The community composition was investigated at locations characterized by biofilm formation and iron-rich bottom pools. Amplicon libraries were sequenced on a MiSeq instrument. The taxonomic survey yielded an unexpected diversity of 25 bacterial phyla including ten genera of iron-oxidizing taxa. The community composition in the snottites and biofilms only slightly differed from the communities found in acidic bottom pools regarding the diversity of iron oxidizers, the key players in most investigated habitats. Sequences of the Candidate Phyla Radiation as, e.g., Dojkabacteria and Eremiobacterota were found in almost all samples. Archaea of the classes *Thermoplasmata* and *Nitrososphaeria* were detected in some biofilm communities.

## Introduction

**Fig. 1:**
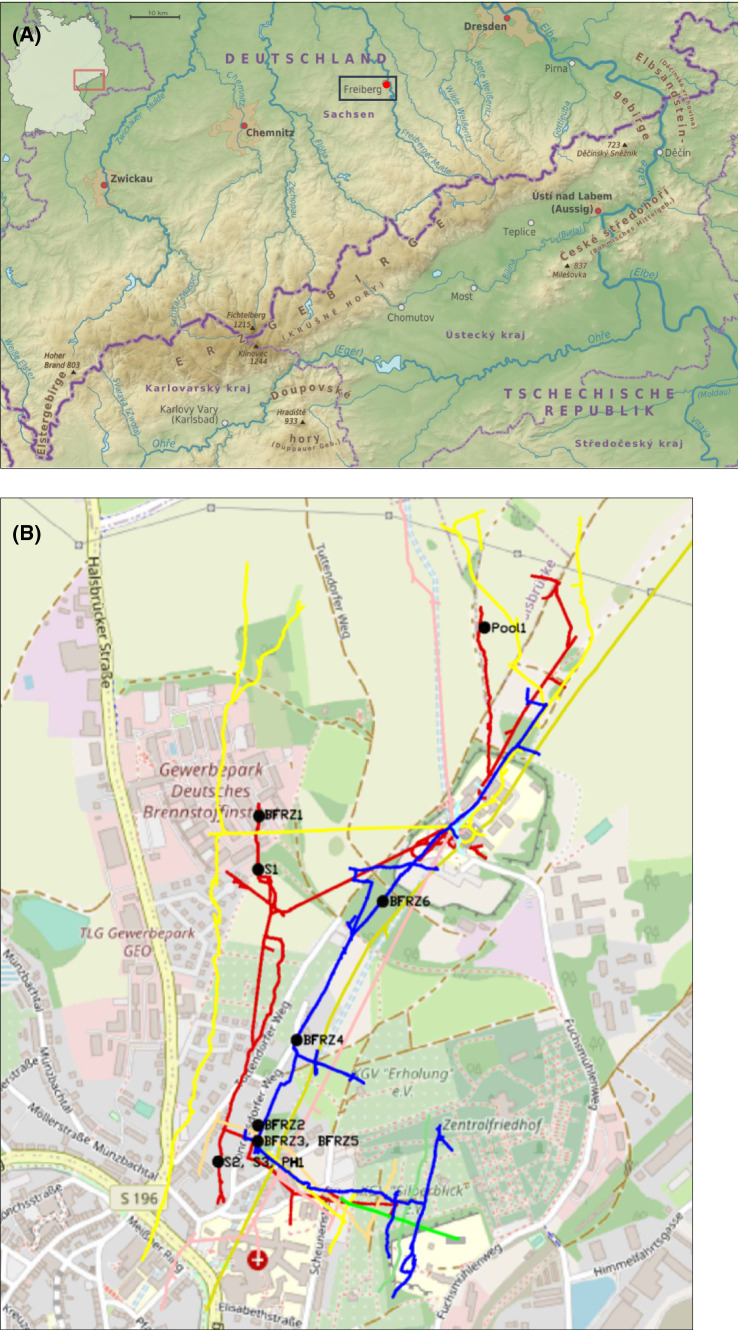
Location of the Reiche Zeche Mine, Freiberg, Saxony at the northern foothills of the Eastern Ore Mountains; image: ore mountains, physical map by Alexrk2 (slightly modified), CC-BY-SA 2.0 (**A**). Overview of the sampling locations on three selected levels within the mine (below ground level), red—285 m, blue—330 m, yellow 260 m (**B**)

The advent of silver mining in the Ore Mountains was initiated by an accidental discovery of solid silver outcrops nearby the village Christiansdorf, the present Freiberg, in 1168. This finding of coveted silver deposits provoked instantly the so-called “first mining clamour” which caused a marked migration of miners from the Harz Mountains. Through history approx. 1100 lodes of the polymetallic sulfide vein-type were explored and mined up to a depth of 800 m (Seifert and Sandmann 2006). The first recorded mention of the Reiche Zeche Mine (RZM) is dated back to the year 1384. Over the centuries the RZM developed on one of the main lodes (Hauptstollngang Stehender) of the central mining district of Freiberg and became the principal mine within a complex network of numerous installations denoted as Himmelfahrt Fundgrube (Fig. 1). Through mining for about 800 years, the district grew to one of the most productive silver mining sites across Europe. 1919 the RZM became an integral part of the Technical University Bergakademie Freiberg. Currently five of the former 17 level driftways are accessible and serve with an extension of 19 km for teaching duties and research assignments (Mischo 2014). In 2020 in association with 21 other Saxon and Bohemian historic mining sites of the Ore Mountains the Freiberg mining district became part of the UNESCO world heritage “Erzgebirge/Krušnohoří Mining Region”.

First observations of the occurrence of chemolithotrophic sulfur- and iron-oxidizing bacteria in mining sites focused on the generation of acid mine drainage (AMD) in coal mines with its attendant environmental problems (Colmer and Hinkle 1947; Temple and Colmer 1951). These studies based on the research of the pioneer microbiologists from the nineteenth century regarding the ‘iron bacterium’ (Silverman and Lundgren 1959). Most of the early studies on microbiologically generated AMD addressed physiological characteristics of pure cultures derived by cultivation from active and abandoned mine sites. While the investigation of biochemical properties of isolates represents an essential part for strain characterization, the exploration of entire microbial communities improves the understanding of ecosystem functioning (Konopka 2009).

Each AMD system consists of a variety of microbial niches that harbor different taxa of an acidophilic community (Baker and Banfield 2003). The species composition of biofilm communities is primarily influenced by geochemical parameters and their variations prevailing on site (Goltsman et al. 2015; Teng et al. 2017). Potential of hydrogen is one of the most relevant factors that determine both, structure and diversity of indigenous communities and relative abundance of dominant taxa within (Kuang et al. 2013; Liu et al. 2014). But also parameters like total nitrogen and iron concentration strongly affect the structure of AMD-influenced communities (Sun et al. 2020). Members of the genera *Acidithiobacillus* and *Leptospirillum* are most commonly found to act as key players in mine-impacted environments at low pH and moderate temperature whether in populations of AMD streams or in biofilm communities (Colmer et al. 1950; Ziegler et al. 2013a). But also, growth and thriving of low abundant species seems to play a crucial role in functional stability of such communities.

Cultivation-independent approaches, like the use of molecular probes and the screening of 16S rRNA clone libraries enable an understanding of key players and dominant taxa within the communities (Edwards et al. 1999; Bond et al. 2000; Bond and Banfield 2001; Tyson et al. 2004). A deeper insight into composition and activity of mine-impacted microbial communities, including rare and underrepresented taxa, greatly benefited from the development of next-generation sequencing systems applied in metagenomics and transcriptomics projects (Amaral-Zettler et al. 2011; Goltsman et al. 2015; Huang et al. 2016). The application of next-generation sequence technologies (NGS) resulted in a great number of metagenomics studies on mine-impacted habitats comprising taxonomic and phylogenetic but also functional topics (Lukhele et al. 2020). Modern sequencing technologies provide access to the rare biosphere and allow to reveal the huge diversity of microbial dark matter, more precisely described as “Candidate Phyla Radiation” (CPR), (Cárdenas et al. 2016; Solden et al. 2016; Hug et al 2016).

To gain a better understanding of the microbial composition with main emphasis on rare members in mine-impacted environments, NGS techniques, enabling a high taxonomic resolution, were combined with appropriate ecological analysis. The study presents the first survey on microbial communities occurring in mine-impacted habitats of the RZM, including the identification of dominant taxa with potential for application in bioleaching of ore from the area (Gelhaar et al. 2015; Schlueter and Mischo 2018).

## Materials and methods

### Site description

The RZM is situated at the northern foothills of the Eastern Ore Mountains (Fig. 1a). The pithead is located at an elevation of 429 m a.s.l. The deepest currently accessible level is the drainage adit Rothschönberger Stolln. It is located at an altitude of ca. 200 a.s.l. regarding the geographical coordinates of the large compound mine “Himmelfahrt Fundgrube”. The ore body mainly consisted of galena, sphalerite, pyrite, chalcopyrite and native silver (Zänker et al. 2002). With 800 years history of ore production the mining operations were ceased in 1969.

### Sampling

Liquid samples were taken from five different locations at 285 m belowground on June 23, 2020. All sampling locations belonged to the tunnel system Wilhelm Stehender Sued, Wilhelm Stehender Nord and Schwarzer Hirsch Stehender. Samples S1, S2 and S3 were taken from slowly flowing AMD; samples PH1 and Pool1 were collected from stagnant AMD (Fig. 1b, red line). Samples were collected into sterile 50 mL Falcon tubes and processed the same day.

**Fig. 2:**
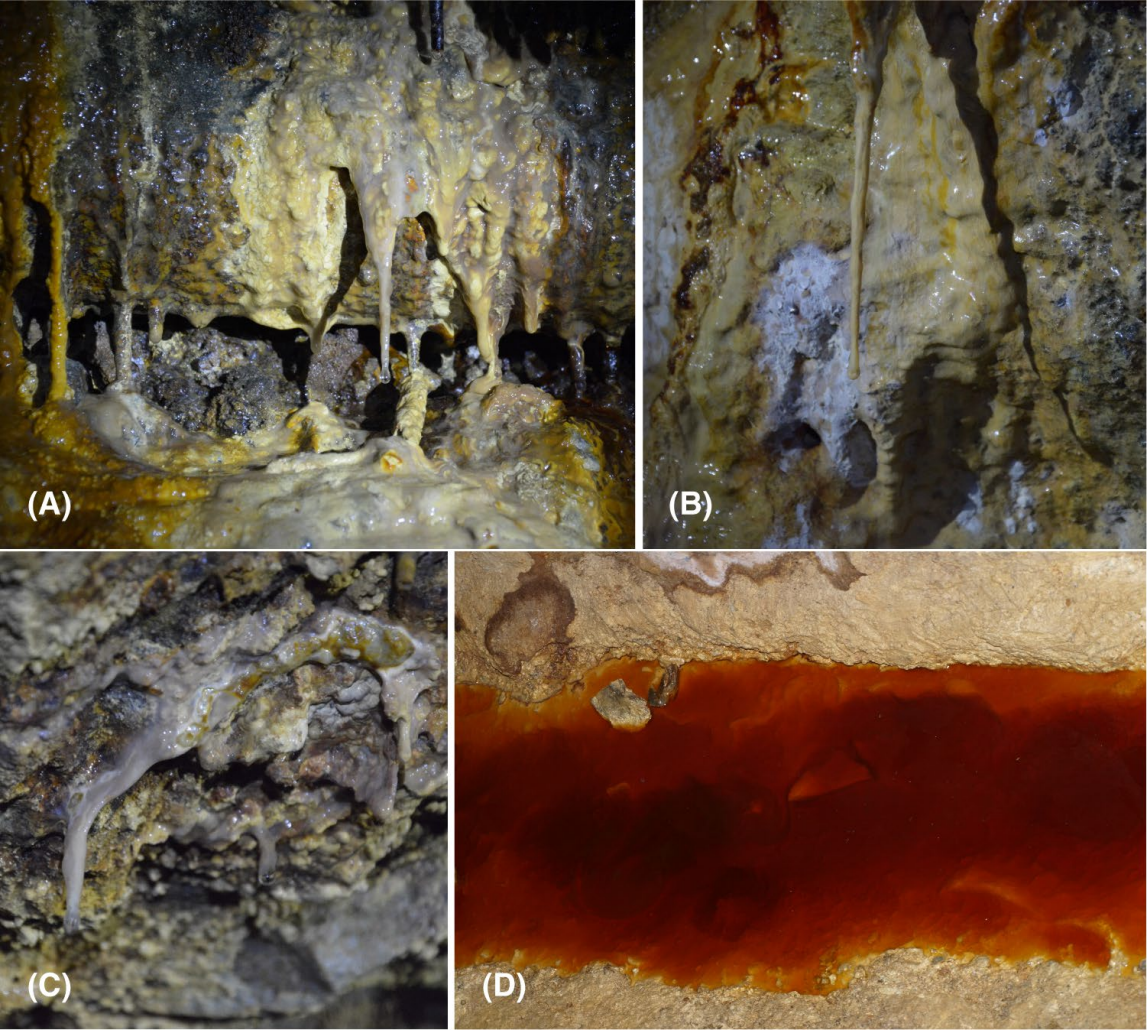
Mat- and snottite-like biofilm formations at sampling location BFRZ1 (**A**), BFRZ2 (**B**) and BFRZ3 (**C**). AMD pools of sampling location S1 (**D**)

Snottite and biofilm samples were collected from six different locations on three underground levels in the mine on November 12, 2020. Samples were taken within the following tunnels: Goldener Frieden Flacher, 260 m belowground; Wilhelm Stehender Sued, Wilhelm Stehender Nord and Schwarzer Hirsch Stehender, 285 m belowground; and Hauptstollngang Stehender, 330 m belowground (Fig. 2). Samples were denoted BFRZ1 to BFRZ6. Fissure water seeping through biofilm structures was collected into sterile 50 mL Falcon tubes for chemical analysis. A total of 20 subsamples were taken from the six selected snottite and biofilm locations. Geographic coordinates for the sampling points are given in Table1. Samples were scraped off from bedrock of sidewalls with sterile spatulas and transferred into sterile 50 mL Falcon tubes; snottites hanging from gallery roofs were cut by sterile scissors. Collected biomass was frozen at − 20 °C until DNA extraction.

**Table 1:** Geographic position of sampling sites

Sampling location	Geographical location		Altitude	Designation	Sampling date
(nos. of subsamples)	Easting	Northing	m.a.s.l		
BFRZ1 (2)	13.351°E	50.929°N	285.3	Wilhelm Sthd. Nord	12.11.2020
BFRZ2 (4)	13.350°E	50.923°N	260.5	Goldener Frieden Fl	12.11.2020
BFRZ3 (5)	13.351°E	50.922°N	332.4	Hauptstollngang Sthd	12.11.2020
BFRZ4 (4)	13.352°E	50.924°N	331.6	Hauptstollngang Sthd	12.11.2020
BFRZ5 (3)	13.351°E	50.922°N	332.4	Hauptstollngang Sthd	12.11.2020
BFRZ6 (2)	13.355°E	50.927°N	330.3	Hauptstollngang Sthd	12.11.2020
PH1 (1)	13.349°E	50.922°N	286.1	Wilhelm Sthd. Sued	23.06.2020
Pool1 (1)	13.358°E	50.932°N	284.1	Schwarzer Hirsch	23.06.2020
S1 (1)	13.351°E	50.928°N	285.1	Wilhelm Sthd. Nord	23.06.2020
S2 (1)	13.349°E	50.922°N	286.1	Wilhelm Sthd. Sued	23.06.2020
S3 (1)	13.349°E	50.922°N	286.1	Wilhelm Sthd. Sued	23.06.2020

### Geochemical analyses

Oxidation reduction potential (ORP), and pH were measured in situ. Temperature and pH were measured using the WTW Sentix 21 pH meter; redox potential vs. Ag/AgCl electrode were determined using the WTW Sentix ORP. Concentrations of minor and major elements were determined by ICP-MS (XSeries 2, Thermo Scientifc) for each AMD and fissure water sample. Calibration solutions (0.01–100 µg l^−1^) were used by adequate dilution of a multi-element stock standard solution (Merck); 10 μg/L rhodium and rhenium served as internal standard following the measuring procedure of Wiche and Heilmeier (2016). The concentration of ferrous iron in AMD and fissure water samples was determined following the Ferrozine method (Braunschweig et al. 2012).

### DNA extraction

Biomass from 50 mL of each AMD-pool sample was collected by centrifugation for 30 min at 12000 ×*g* and 4 °C. Total DNA was extracted from snottite, biofilm, and AMD samples using the Power Soil DNA Isolation Kit (MoBio Laboratories, Carlsbad, USA) following the manufacturer’s instructions. Quality and quantity of purified DNA was determined spectrophotometrically (NanoDrop 1000 Spectrophotometer, Thermo Scientific, Waltham, MA, USA). Additionally, DNA quality was checked by electrophoresis on a 0.8% agarose gel. DNA was stored in sterile water at − 20 °C prior to analysis.

### 16S rRNA library preparation and sequencing

The 16S rRNA universal primers 341F (5′-CCTACGGGNGGCWGCAG-3′) and 805R (5′-GGACTACHVGGGTATCTAATCC-3′) were extended by the specific Illumina adapter overhang sequence, and used for the amplification of the highly variable V3/V4 region in prokaryotic genomes (Hugerth et al. 2014; Takahashi et al. 2014). The V3/V4 region is considered as one of the most informative regions for NGS-based metagenomic studies. Use of the selected primers is recommended for 16S metabarcoding studies since they target the domains bacteria and archaea on a broad scale (Klindworth et al. 2013). Sequencing libraries were labeled with multiplex indexing barcodes using the Nextera XT Index Kit (Illumina, San Diego, USA) following manufacturer’s recommendations. The PCR reaction was carried out in a 25 µL reaction volume with 12.5 µL2 × KAPA HiFi HotStart ReadyMix (KAPA Biosystems, Wilmington, MA, USA), 0.2 µM final concentration of each primer, and 12.5 ng template DNA. Thermal cycling consisted of denaturation at 95 °C for 3 min, followed by 25 cycles of 95 °C for 30 s, 55 °C for 30 s, and 72 °C for 30 s, and finally 72 °C for 5 min. Quantity and quality of the libraries were assessed applying the Qubit™ 1X dsDNA HS Assay Kit (Thermo Fisher Scientific). The libraries were sequenced on a MiSeq platform utilizing the v3 reagent kits (Illumina, San Diego, USA).

### Sequence processing and analyses

Sequence reads were subjected to quality control by FastQC prior processing (Wingett and Andrews 2018). Sequences were processed by Mothur (v.1.37.4) following the related standard operating procedure (Schloss et al. 2009). Minimum sequence lengths were set to 400 bp; the minimum overlap length for the contig assembly was set to 50 bp. A maximum of seven homopolymers was allowed. Chimeric sequences were identified and subsequently removed using the VSEARCH algorithm within Mothur. Sequences were aligned to the SILVA alignment database (SILVA SSU v.132) for clustering and creating the distance matrix (Pruesse et al. 2007; Quast et al. 2013). Pairwise distances with a cutoff value of 0.03 were calculated using the Needleman-Wunsch alignment algorithm (Needleman and Wunsch 1970). Classification of sequences and operational taxonomic units (OTUs) was done with SILVA alignment database likewise. The sequencing error rate was calculated by including the staggered, low concentrated HM-783D microbial mock community serving as standard (BEI-Resources, Manassas, U.S.A.).

### Statistical analyses

Alpha diversity measures were calculated within Mothur. Calculations based on the absolute number of sequences per OTU; the alpha diversity measurements included the total number of observed OTUs, the estimated number of OTUs that could be detected if every individual OTU was detected (ACE richness estimator). The Shannon’s diversity index H′ was calculated as qualitative measurement of equivalents to species numbers in the corresponding habitat. The inverse Simpson’s index was included as it puts less emphasis on the rare OTUs and may give a clearer illustration of diversity for the investigated communities compared to the Shannon–Wiener diversity index. The Evenness index describes the pattern of relative species abundances in a community. The Shannon evenness is a diversity index, which in contrast to Simpson evenness provides information on the species equitability unaffected from species of high dominance in the community. Hence it seems useful to calculate both evenness indices. The Berger–Parker index was calculated as robust measure for the dominance of the most common OTU in each community. The (dis)similarity of communities was calculated by ThetaYC sample metrics based on a 0.03 OTU distance cutoff. Hierarchical clustering was visualized as heatmap and dendrogram.

## Results

### Geochemical analysis

The temperature at all sampling locations was constant at 12 °C. The pH varied between 1.6 (Pool1) and 2.6 (S1) for the AMD samples and 1.9 (BFRZ1) and 2.6 (BFRZ6) for the biofilm (including snottite) samples, respectively. The ORP measured at the AMD sampling locations (vs. Ag/AgCl) varied from 493 mV (S3) to 645 mV (Pool1). AMD sample S3 and fissure water of BFRZ1 contained the highest concentration of iron whereas AMD sample Pool1 exhibited the highest concentration of Mn, Ni, Zn, Cd, and Pb. The biofilm sample BFRZ1 exhibited elevated concentrations especially for the (heavy) metal cations: Al, Cr, Mn, Co, Ni, Cu, and Zn. Overall, AMD sample S1 contained the lowest concentrations of all measured elements. The elemental composition of fissure waters from BFRZ samples displays strong heterogeneity. Physicochemical parameters and a summary of the elemental analysis from AMD and fissure water samples are given in Table 2.

**Table 2:** Hydrochemical parameters of six biofilm and five AMD-pool locations

	BFRZ1	BFRZ2	BFRZ3	BFRZ4	BFRZ5	BFRZ6	PH1	Pool1	S1	S2	S3
Physicochemistry											
pH	1.9	2.1	2.0	2.5	2.3	2.6	2.4	1.6	2.6	2.5	2.1
Temperature	12 °C										
E_h_ (mV)	591	520	506	487	480	465	536	645	566	470	493
Major elements (g/L)											
S (DTS)	1.6	1.1	0.5	0.3	0.5	0.4	3.2	2.0	1.0	8.8	21.2
Fe_total_ / (Fe^2+^)	10.2 (0.23)	1.2 (1.0)	0.3 (3.9 * 10^–3^)	0.1 (0.6 * 10^–3^)	0.3 (1.9 * 10^–3^)	0.1 (0.1 * 10^–3^)	9.7 (LDL)	17.3 (LDL)	0.2 (0.01)	3.6 (0.11)	21.6 (0.59)
Zn	4.7	0.3	0.3	0.1	0.2	0.1	19.1	8.5	0.2	3.9	11.3
Minor elements (mg/L)											
Ni	5.4	0.1	0.3	0.2	0.2	0.6	32.4	5.6	0.3	9.3	23.9
Cu	374.3	1.6	3.6	1	1	1.3	323.6	315.1	3.9	94.9	352.7
Cd	3.5	2.4	1.9	0.8	1.3	0.4	119.8	75.5	1.5	26.1	74.7
Pb	0.8	1.9	1.5	1.8	1.7	0.4	293.3	0.2	0.2	14.8	246.1
Mn	0.95	0.06	0.05	0.05	0.04	0.06	8604	722	68	1068	1696
As	325	85	21	4	7	2	3700	211	0.4	321	6297

Sulfur determined as DTS (dissolved total sulfur)

### Summary sequencing data

**Fig. 3:**
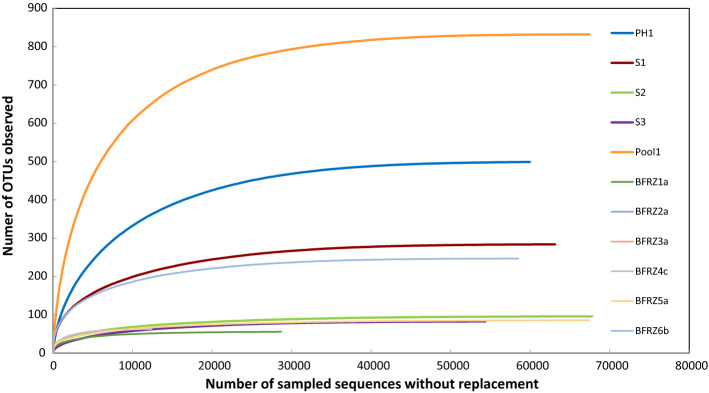
Rarefaction analysis for observed OTUs at 97% sequence similarity (approximation to species level) for the five AMD-pool and six representative biofilm samples

Sequencing the V3/V4 amplicon libraries on a MiSeq platform yielded a total of 2,646,937 reads for the 25 datasets. Therefrom a number of 1,306,610 contigs with read lengths between 427 and 453 bp could be generated. 6013 chimeric sequences were found and eliminated. After clustering, removal of singletons and elimination of chimera, 4758 unique sequences (comprising 1,211,112 sequences in total) were retrieved and used for classification on genus level. A total of 21,463 OTUs were generated after clustering at a 97% similarity level (approximation to species resolution). The number of OTUs per sample ranged from 34 (BFRZ2d) to 832 (Pool1). The rarefaction curves in Fig. 3 approach an asymptote for all 25 samples, suggesting that the microbial phylotypes present in each sample were identified almost completely. This was confirmed by the high Good’s coverage, reaching almost 100%, for all samples (Tab. 3). These results provide an estimate of sampling completeness. The error rate of 0.021% was calculated on the basis of the sequenced mock community and corresponded well with test results (Sze and Schloss 2019).

**Table 3:** Alpha diversity metrics for the prokaryotic community of the investigated habitats at Reiche Zeche Mine

	Nos. sequences	Good's coverage (%)	S_obs_ (OTU richness)	ACE richness estimator	Shannon index	Simpson (Inv) index	Shannon evenness	Simpson evenness	Berger-Parker index
AMD-pool									
communities									
PH1	59,966	99.9	499	506	2.27	3.19	0.36	0.01	0.53
S1	63,133	99.9	284	289	3.42	17.61	0.61	0.06	0.12
S2	67,792	99.9	96	98	1.52	2.89	0.33	0.03	0.53
S3	54,376	99.9	83	87	1.42	2.62	0.32	0.03	0.58
Pool1	67,436	99.9	832	840	3.87	11.37	0.58	0.01	0.22
Biofilm									
communities									
BFRZ1a	28,568	99.9	56	57	1.48	2.51	0.37	0.05	0.59
BFRZ1b	24,664	99.9	68	68	1.53	2.47	0.36	0.04	0.61
BFRZ2a	36,134	100	64	64	2.56	8.40	0.62	0.13	0.24
BFRZ2b	36,039	99.9	41	41	1.29	2.27	0.35	0.06	0.63
BFRZ2c	45,820	99.9	36	37	1.00	1.85	0.28	0.05	0.72
BFRZ2d	30,775	100	34	34	1.56	3.18	0.44	0.09	0.51
BFRZ3a	36,913	99.9	97	97	2.87	8.51	0.63	0.09	0.29
BFRZ3b	31,680	99.9	116	118	2.85	8.79	0.60	0.08	0.26
BFRZ3c	56,056	99.9	107	112	2.01	4.99	0.43	0.05	0.33
BFRZ3d	58,766	99.9	74	77	1.50	2.94	0.35	0.04	0.47
BFRZ3e	65,954	99.9	74	76	2.16	5.86	0.51	0.08	0.28
BFRZ4a	32,808	100	108	111	3.02	12.04	0.65	0.12	0.18
BFRZ4b	56,520	100	93	97	2.04	4.56	0.47	0.06	0.38
BFRZ4c	12,425	99.9	63	64	2.11	4.77	0.51	0.08	0.37
BFRZ4d	48,537	99.9	111	111	2.75	9.07	0.60	0.09	0.22
BFRZ5a	67,494	99.9	86	87	2.00	4.51	0.45	0.05	0.37
BFRZ5b	53,648	99.9	109	109	2.94	10.86	0.63	0.10	0.20
BFRZ5c	63,039	99.9	108	108	3.03	13.79	0.65	0.13	0.14
BFRZ6a	54,090	99.9	197	197	2.50	4.07	0.47	0.02	0.48
BFRZ6b	58,440	99.9	247	248	3.28	11.47	0.60	0.05	0.24

Calculations based on OTUs at 3% cutoff level (approximation to species level)

### Alpha and beta diversity in AMD, snottite, and biofilm samples

A total number of 312,742 sequence reads were received for the AMD samples after sequence trimming and processing. The sequence number per sample varied from 54,376 in sample S3 to 67,470 in sample Pool1 (Tab.3). The mean number of OTUs and ACE estimated number of OTUs differed widely between the samples, with the lowest numbers of 83 OTUs and 87 ACE estimated OTUs observed for S3 and the highest numbers of 832 OTUs and 840 ACE estimated OTUs detected in Pool1. Shannon’s diversity index H′ was lowest, H′ = 1.42, in S3 and highest, H′ = 3.87 in Pool1. A saturated sampling depth is reflected by Good's coverage of at least 99.9% for all samples.

The number of sequence reads detected in the biofilm (including snottite) samples varied from 12,425 sequences in BFRZ4c to 67,494 in BFRZ5a, with a total number of 898,370 sequences for all 20 samples (Table 3). The mean number of OTUs and ACE estimated number of OTUs was 94 and 96, respectively, with the lowest numbers of 34 OTUs for both, Sobs and ACE in sample BFRZ2d. The highest numbers of 247 OTUs (Sobs) and 248 ACE estimated OTUs were detected in sample BFRZ6b. Shannon’s diversity index H′ was lowest, H′ = 1.00, in BFRZ2c and highest, H′ = 3.28, in BFRZ6b. Good's coverage values indicated that not less than 99,9% of the total species richness was accounted for each sample.

**Fig. 4:**
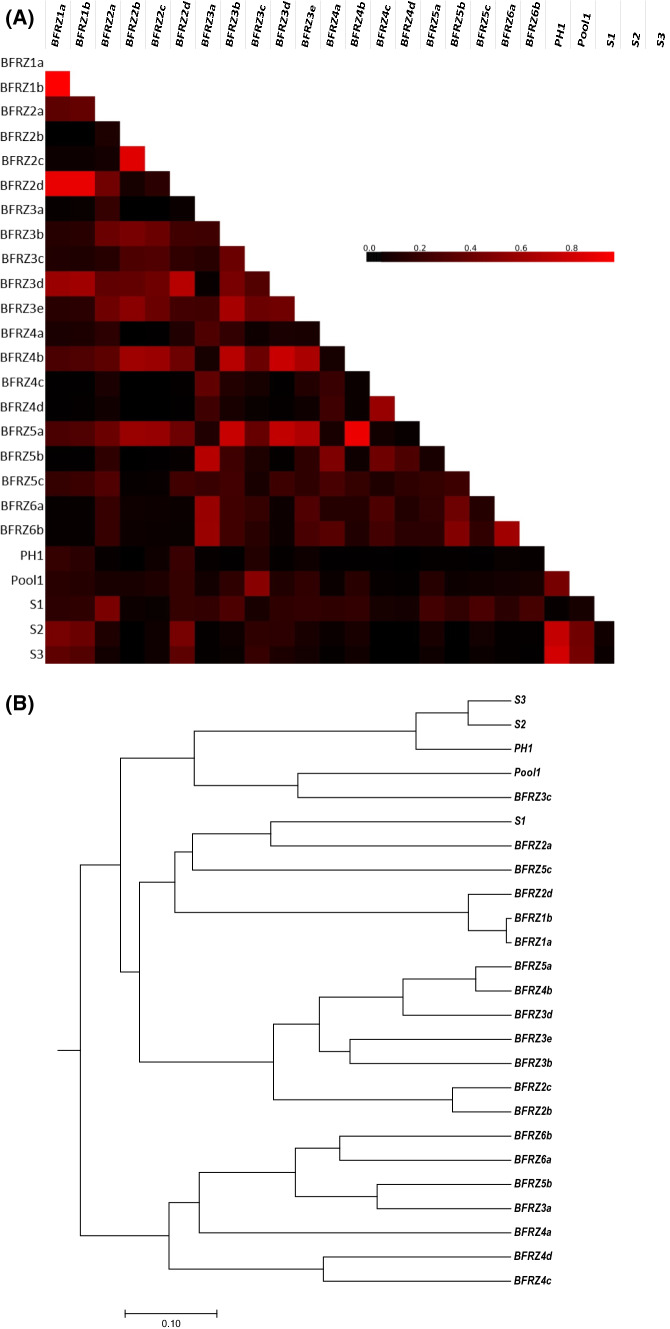
Beta diversity analysis to estimate dissimilarity of bacterial community compositions among the 25 samples. **A** Heatmap derived from dissimilarity matrix of ThetaYC distance. **B** UPGMA dendrogram representing the relatedness of prokaryotic communities from each sampling location based on the OTUs defined at a 0.10 distance cutoff. The relationships amongst samples are displayed based on the ThetaYC similarity. BFRZ1a to BFRZ6b represent every subsample from each biofilm sampling location; PH1 and Pool1 depict samples derived from stagnant AMD, S1 to S3 from flowing AMD

**Fig. 5:**
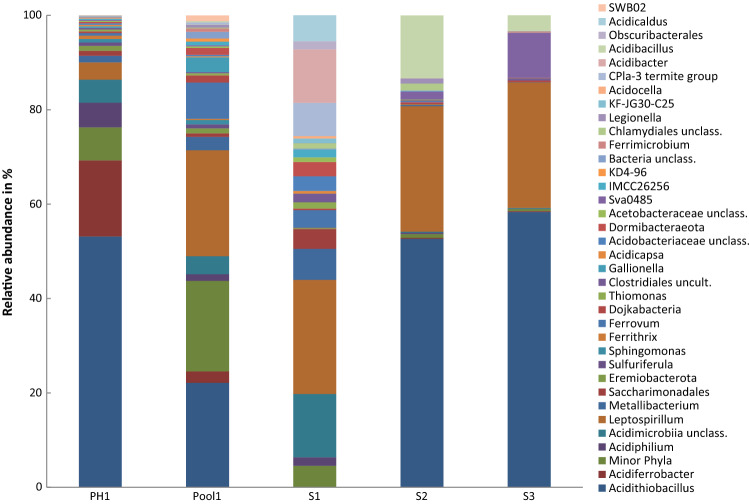
Taxonomic distribution of prokaryotic communities in AMD-pool habitats. The relative abundance is assigned to the corresponding genus, based on SILVA SSU v.132 database. Taxa with low abundance (< 0.5%) are combined and displayed as “Minor phyla”

**Fig. 6:**
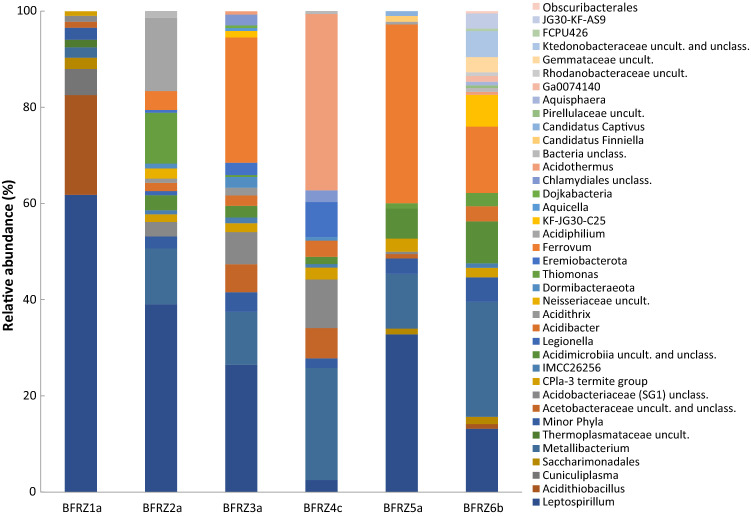
Taxonomic distribution of prokaryotic communities in selected biofilm habitats; one example from each location. The rel. abund. is assigned to the corresponding genus, based on SILVA SSU v.132 DB. Taxa with low abund. (< 0.5%) are combined and displayed as “Minor phyla”

The relationships between all communities were analyzed based on Yue and Clayton theta distances; the similarities based on the OTU composition. To facilitate the analysis, a heatmap and a clustering dendrogram were constructed. The biofilm (including snottite) communities were generally distinct from AMD-pool communities (with the exception of subsample S1) according to the analysis of the clustering dendrogram (Fig. 4B). The community compositions of both habitat types show only little difference in the diversity of the ferrous iron-oxidizing subpopulations (Figs. 5 and 6). Based on the OTUs at a cutoff of 0.10, the similarities of communities ranged from 0.1% (samples from different locations as, e.g., BFRZ4c in relation to S3) to 98% (subsamples from the same location, e.g., BFRZ1) (heatmap see Fig. 4A).

### Archaeal signatures

16S rRNA gene sequences from archaea were obtained from three of the 20 biofilm samples, i.e., BFRZ1a, BFRZ1b and BFRZ6b. The relative abundance of sequences for the genus *Cuniculiplasma* was highest in BFRZ1a with 5.4%. The uncultured *Thermoplasmatales*, denoted ‘E-plasma’, were present in samples BFRZ1a and BFRZ1b at a relative abundance below 0.5%. Sequences of acidophilic, ammonia-oxidizing *Candidatus* Nitrosotaleaceae were detected only in sample BFRZ6b likewise with a relative abundance below 0.5%. For the five investigated AMD communities no archaeal sequences were retrieved.

### Bacterial signatures

In total, 60 bacterial genera from 25 phyla with a relative abundance of at least 0.5% of the sequence reads were detected in the samples. Autotrophic iron-oxidizing bacteria constituted the group of core genera by covering ten genera, contributing with 8–93% (BFRZ4c, S2) of the total number of sequence reads in all samples. The proportion of FeOB within the bacterial communities of the biofilm samples was in average lower than in the AMD samples. Sequences of the genus *Leptospirillum* were ascertained in all 25 samples; relative sequence abundance ranged from 3 to 52%. *Acidithiobacillus* could be detected in all samples except sample BFRZ5a; the relative abundance varied from below 0.5% (minor phyla) to 58% (Fig. 5). The taxon *Ferrovum* was present in 21 samples and reached relative abundances of up to 72% (BFRZ2c).

Sequences of heterotrophic FeOB occurred in most of the samples. Within the phylum *Actinobacteria* sequences of iron-oxidizing *Acidimicrobiia* (including the genera *Acidithrix* and *Ferrimicrobium*) were present in all samples except BFRZ2c and AMD samples S2 and S3. The relative abundances varied between 0.2 (BFRZ3d) and 13.4% (S1) of the sequences. The genus *Acidibacillus* reached relative abundances of up to 13.3% as in sample S2.

The relative abundance of iron-reducing bacteria (FeRB) was not remarkably lower than that of the FeOB in most of the samples. Sequences of FeRB were found in all three sample types, biofilm, snottite and AMD. Iron-reducing *Acidibacter* was present at relative abundances of 0.5 (BFRZ2b) to 13.3% (S2) in twelve of the 25 samples. Members of the family *Acidobacteriaceae*, including the genera *Acidicapsa* (S1) and *Acidipila* (BFRZ5b) were present in eleven samples at a relative abundance of 0.5 (BFRZ5a) to 22.0% (BFRZ4d). Sequences belonging to the genus *Clostridia* were detected in four samples contributing to the communities at relative abundances of 0.5 (BFRZ5b) to 3.4% (BFRZ3a). Sequences of *Acidiphilium* species were widely common in biofilm and AMD samples, relative abundances in eleven samples ranged from 0.5 (BFRZ3e) to 15.2% (BFRZ2a) (Fig. 6). Numerous sequences of the family *Acetobacteraceae* could not be affiliated on genus level but were prominent in most of the samples displaying relative abundances from 0.5% (BFRZ2a) to 7.5% (BFRZ4a). Sequences of *Metallibacterium* were detected at relative abundances of 0.1 (BFRZ2c) to 47.9% (BFRZ6a) in all samples except AMD samples S1, S2 and S3.

Bacteria of the yet uncultured CPR were present with at least one taxon in all samples at relative abundances of between 0.1% (candidate phylum Dormibacteraeota, in BFRZ2c) and 14.1% (*Candidatus* phylum Eremiobacterota in BFRZ4d) except in sample BFRZ2d. The *Candidatus* phylum Dependentiae (formerly TM6) and the candidate superphylum Patescibacteria occurred in all AMD samples and in the snottite samples of BFRZ3 to BFRZ6. Sequences of *Candidatus* phylum Dojkabacteria (formerly WS6) were found in all AMD-pool samples with a relative abundance of up to 1.5%; the phylum was also detected in biofilm sample BFRZ1b. Sequences of candidate phylum Latescibacteria (formerly WS3) occurred only in AMD sample Pool1.

## Discussion

The Reiche Zeche Mine, created from the merger of the two historic mines “Vordere Reiche Zeche” and “Hintere Reiche Zeche”, belongs to the oldest facilities of the mining landscape in the Ore Mountains. The mine waters seeping through rocks and spreading along tunnels are commonly acidic and can be categorized as extreme environment at many mine locations according the definition for an optimal life of extremely acidophilic microorganisms at pH below 3.0 (Johnson and Quatrini 2020). Generally, the mine waters contain high amounts of iron, zinc, arsenic, many other metal(loid)s and sulfate. Iron was found to be one of the dominant parameters controlling metal presence in both, stagnant and flowing drainage waters in the RZM (Zhiteneva, et al. 2016). However, elemental analyses of mine drainage samples taken from several locations showed strong distinctions in the chemical composition.

The prokaryotic communities detected in the snottite, biofilm, and AMD samples consisted largely of aerobic organisms, although anaerobic types capable of either fermentation or anaerobic respiration on ferric iron were present as well, although in lower abundance. There were no indications for sulfate reduction in the potentially anaerobic micro-niches of the snottites or biofilms. However, in four of the five AMD-pool habitats sequences affiliated with the Candidate Sva0485 clade (class *Deltaproteobacteria*) were found to occur up to a relative abundance of 9.5% as in sample S3. Clade Sva0485 is known to be present in different AMD ecosystems and to contain potential sulfate-reducing bacteria (Ayala-Muñoz et al. 2020; Vuillemin et al. 2018). The ecological role of this taxon remains still unknown due to the lack of physiological and genomic insights (Tan et al. 2019).

In most of the habitats the communities are clearly dominated by autotrophic FeOB commonly acting as primary producers in acidic mine-impacted environments. In numerous studies on microbial communities in such habitats, *Acidithiobacillus* has been shown to operate as key taxon, as, e.g., it accelerates habitat acidification by generation of sulfuric acid; works as biofilm architect by releasing extracellular polymeric substances (EPS) and supports growth of heterotrophs by the release of small organic substances (Jones et al. 2011; Karavaiko and Pivovarova 1973). In many of the individual habitats, members of the genus *Leptospirillum* are similarly dominant as *Acidithiobacillus*. They are found in high abundance in mine-impacted biofilm communities (Bond et al. 2000; Wilmes et al. 2009). The biochemical basis for biofilm formation was shown through genome analysis of the model species *L. ferriphilum* (Tyson et al. 2005; Goltsman et al. 2009). Growth and thriving of microbial communities in the commonly oligotrophic and nitrogen deficient mine-impacted environments not only depend on pioneer organisms capable of carbon fixation, as a prerequisite for microbial colonization also nitrogen fixation is required (Johnson and Hallberg 2008). The nitrogenase gene (nifH) was detected in a number of *Leptospirillum* isolates already physiologically described as diazotrophic organisms. Consequently, it is assumed that in many AMD habitats *Acidithiobacillus ferrooxidans* but also members of *Leptospirillum* and *Ferrovum* act as key species for nitrogen fixation (Levicán et al. 2008; Goltsman et al. 2009; Johnson et al. 2014).

In all samples pH values varied between 1.6 (Pool1) and 2.6 (BFRZ6). This pH range is suitable for growth of a variety of extreme acidophiles. The formation of extremely acidic habitats in mining environments requires the presence of sulfur-oxidizing bacteria (SOB). At some sampling locations the relative abundance of SOB other than *Acidithio-bacillus* are noticeably high. Sequences of the facultative chemolithoautotroph *Thiomonas* make up 16 and 20% relative abundance in samples BFRZ2b and BFRZ3e, respectively. The predominant occurrence of *Thiomonas* in Fe-As-rich AMD habitats was shown, e.g., for the mining effluents of Carnoules, displaying arsenic concentrations comparable to those of RZM (Bruneel et al. 2011). The mixotrophic *Sulfuriferula*, recently reclassified from the genus *Thiobacillus*, was found in two of the AMD samples as one of the minor SOB (PH1 and Pool1). *Sulfuriferula* is considered as sulfur oxidizer in moderately acidic habitats and was described as potential key player in sulfur cycling in mine waste (Jones et al. 2017).

The investigated mine environment is characterized by oxic drainage waters. Chemical weathering occurs through contact of the exposed ore-containing rock surfaces with oxygen and water. A mineralization of predominantly sulfidic ores like pyrite, galena, sphalerite, chalcopyrite, and arsenopyrite was found to be characteristic for the polymetallic deposit (hydrothermal Pb–Zn–Ag type) of “Himmelfahrt Fundgrube” (Seifert and Sandmann 2006; Stockmann et al. 2013). The release of iron and sulfate from the host rock leads to a pH decrease of the mine water. The presence of reduced iron and oxygen in the acidic mine waters strongly influences the composition of microbial communities dwelling in these environments. In the RZM the microbial communities consist mainly of acidophilic iron- and sulfur-oxidizing bacteria. Almost all taxa of the FeOB and SOB found in the 25 sampled mine locations thrive in oxic environments and contribute to AMD formation. The increase in acidity in conjunction with the generation of oxidative ferric iron leads to a progressing rock dissolution.

An unexpected high prokaryotic diversity was found in all three sample types, biofilm, snottite and AMD collected from three different levels in the RZM. A Shannon index of H` ≥ 3.0 was found for the community diversity at ten locations, reaching the highest diversity at Pool1 (H` = 3.9). This is an interesting finding, since high prokaryotic diversity seems uncommon for most AMD habitats upon these pH conditions (Teng et al. 2017). Most commonly, lower community diversity is found at locations influenced by lower pH (Kuang et al. 2013).

In all investigated samples the number of detected genera displaying a relative abundance ≥ 0.5% varied between 4 (sample BFRZ2c) and 23 (BFRZ6b). The richness on species level obtained using the ACE estimator showed variations between 34 (BFRZ2d) and 838 (Pool1). Due to the removal of singletons during sequence processing the commonly used Chao1 calculator for richness estimation could not be applied in the alpha diversity analysis. Moreover, as rarefaction analysis already displays, an almost complete detection of occurring taxa can be expected and ACE estimation is unlikely to differ strongly from the number of observed OTUs.

**Fig. 7:**
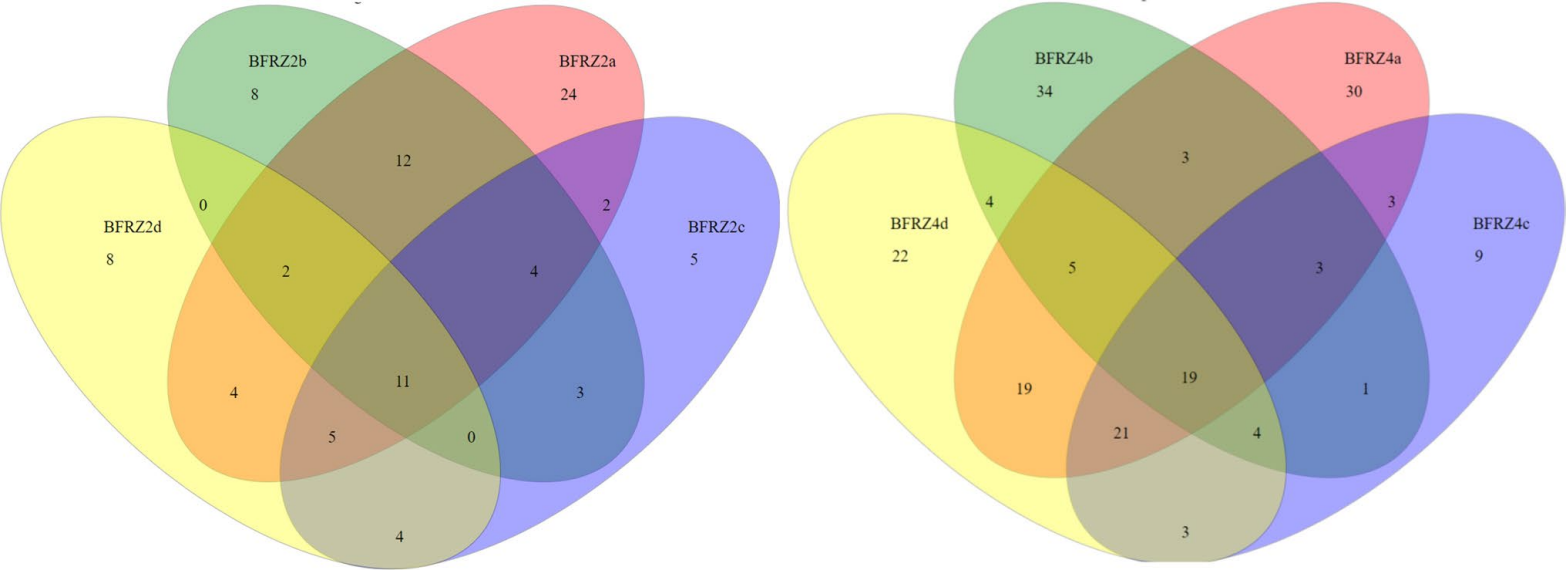
Summary of shared and unique OTUs at 3% distance threshold for biofilm communities. Subsamples taken at locations BFRZ2, and 4 shown as representatives for all investigated biofilm locations

**Fig. 8:**
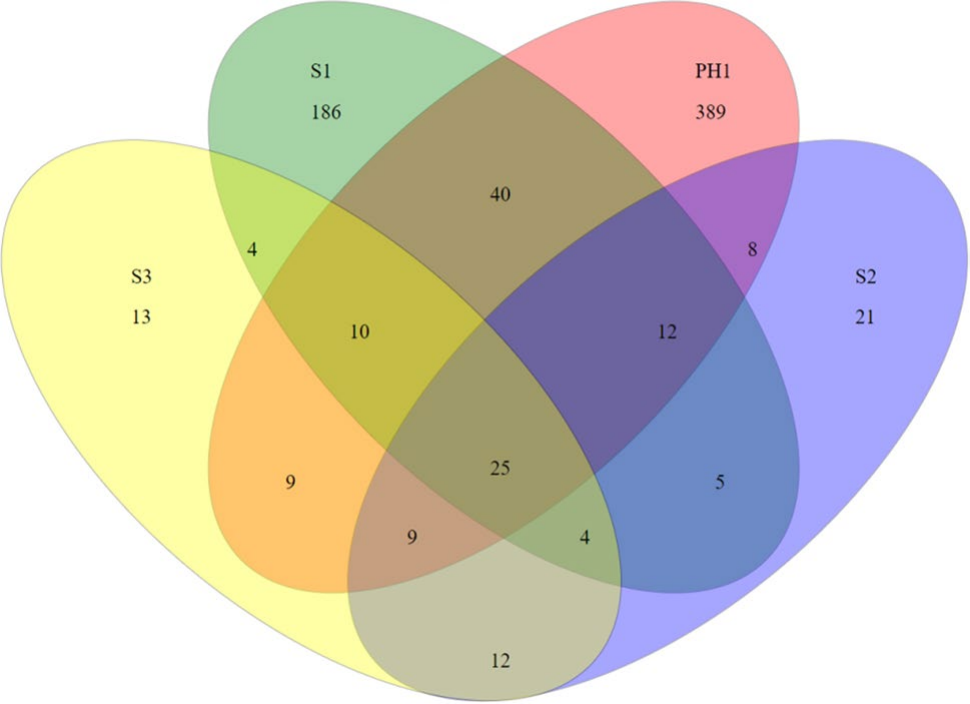
Summary of shared and unique OTUs at 3% distance threshold for the AMD-pool habitats PH1 and S1–S3

In Fig. 7 the Venn diagram depicts the number of OTUs unique to the respective subsample or shared among multiple subsamples. The OTU analysis indicated that 11 OTUs were common to all subsamples at location BFRZ2; the numbers of unique OTUs are lower with the exception of subsample BFRZ2a. This ratio of unique to shared OTUs reflects a more or less similar composition of the communities at sampling location BFRZ2. At sampling location BFRZ4, in contrast, the unique OTUs of each subsample (except BFRZ4c) exceed the shared OTUs. Here the communities across all subsamples seem to be rather heterogeneously composed. In Fig. 8, the Venn diagram presents a different picture for the microbial communities of the AMD pools. The number of unique OTUs range from 13 (S3) to 389 (PH1). The wide span of OTUs obtained for these communities impedes a direct comparison of subsamples for diversity analysis at OTU level.

From the results of beta diversity analysis (Fig. 4A, B), it can be revealed that distances between the communities of different locations are as large as distances between subsamples from single sampling locations. Presumably, environmental factors change on a small spatial scale, thereby causing the development of communities with diverse taxonomic composition in close vicinity. Comparable observations were described from related mine-impacted habitats (Liang et al. 2017). Similar phenomena were observed when the distances of biofilm and AMD-pool communities were compared. As the cluster analysis in Fig. 4B depicts, there are only minor dissimilarities in the microbiome structure of both habitat types (see for instance topology of community BFRZ3c in the dendrogram).

Communities in mine-impacted habitats are often dominated by only few taxa, such as *Acidithiobacillus*, *Leptospirillum*, and *Ferrovum* (Baker and Banfield 2003; Hallberg et al. 2006; Brockmann et al. 2010). This is in agreement with the results presented in this study. The dominance of *Acidithiobacillus* was mostly found in the AMD samples, whereas *Leptospirillum* and *Ferrovum* predominantly occurred in snottites and biofilms.

The occurrence of heterotrophic, iron-reducing bacteria like, e.g., *Acidiphilium* and *Metallibacterium* provides an indication for the occurrence of anoxic niches and microhabitats inside acidophilic biofilms as it was described for biofilms of the pyrite mine “Drei Kronen und Ehrt” (Ziegler et al. 2013a). The two genera were detected in almost all of the samples from the RZM. With 48%, the relative abundance of *Metallibacterium* is remarkably high in sample BFRZ6a. Simultaneously, the pH for this location is highest among all sampling points (pH 2.6). The species *M. scheffleri* was shown to have an alkalinizing effect on the milieu by ammonia release under certain growth conditions (Ziegler et al. 2013b). Consequently, it may be possible that less acidic micro-niches can be generated even in an environmental setting with an overall pH below 2.5, if *Metallibacterium* is present in the biofilm. The provision of a favorable pH microenvironment by *Metallibacterium* might be the reason for the occasional occurrence of much less acidophilic *Legionella* in AMD-influenced snottites and biofilms, as, e.g., in sample BFRZ6a. The phenomenon of *Legionella* being present in acidic biofilm communities has been investigated in detail for habitats from Yellowstone National Park (Sheehan et al. 2005).

In contrast to several other worldwide investigated mining habitats of comparable geochemical conditions the prokaryotic communities in the RZM contained archaeal populations at only two locations (BFRZ1 and BFRZ6). Sequences of the genus *Cuniculiplasma* were found with a relative abundance of 5.4% at sampling location BFRZ1a. The cell-wall deficient, facultative anaerobic organism is known from various mining environments as, e.g., in south-west Spain and North Wales and was shown to use sulfur as electron acceptor like other members of the *Thermoplasmatales* (Golyshina 2016; Segerer et al. 1988). Members of the *Thermoplasmatales* are frequently located in mine-impacted communities where they seem to be involved in the degradation of biofilm components (Chen et al 2016). The biofilms at location BFRZ1 are fed by high sulfur concentration in the fissure water (1.6 g/L). Since most of the sulfur in the seeping mine waters occurs as sulfate, but sulfate reducers could not be retrieved in the biofilm community, it seems likely, *Cuniculiplasma* depends on intermediate sulfur species generated during the oxidation of sulfide-containing ores. The obligatory use of polypeptides as growth substrate makes a later appearance of *Cuniculiplasma* during biofilm succession likely. A share of 1.6% from the community of sample BFRZ1a was identified as *Thermoplasmataceae* but could not be affiliated on genus level. Likewise, sequences belonging to the phylum *Thaumarchaota* in sample BFRZ6b could not be specified further than up to the ammonium oxidizing *Candidatus* Nitrosotaleaceae (part of minor phyla). The occurrence of this Candidatus family was shown in mine-impacted habitats and is discussed as important group for nitrification in low pH environments (Herbold et al. 2017; Gavrilov et al. 2019; Miettinen et al. 2021).

Although the pH values range from 1.6 to 2.6 for all investigated habitats, a distinct tendency towards higher diversity in the less acidic environments as described for, e.g., microbial communities in mine tailing impoundments could not be observed (Korehi et al. 2014; Liu et al. 2014).

Interestingly, from the AMD location Pool1 sequences of the genus *Gallionella* could be retrieved with a relative abundance of 3.2%. The pH at this site was 1.6 and thereby not in the range to expected for the common occurrence of *Gallionella*, an FeOB primarily known from circumneutral, microaerophilic and often ochre-rich watercourses (Emerson and Moyer 1997). However, the distribution of *Gallionella* was also reported from mine-impacted communities at pH ≤ 3 (Hallberg et al. 2006; García-Moyano et al. 2015; Heinzel et al. 2009). The interplay of pH and ferrous iron concentration has been described as suitable indicator for the prediction, if genera like *Gallionella* (pH > 3, Fe^2+^ > 4 mM), *Acidithiobacillus* (pH < 3, Fe^2+^ < 4 mM) or *Ferrovum* (pH < 3, Fe^2+^ > 4 mM) are likely to occur in the investigated habitats (Jones et al. 2015). However, the habitats of RZM do not fit entirely this categorization.

Under oxygen-limited conditions several auto- and heterotrophic acidophiles like, e.g., some *Acidithiobacillus* species, *Metallibacterium*, *Acidiphilium*, *Acidobacterium*, and *Acidibacter* can utilize ferric iron as terminal electron acceptor instead of oxygen. Interestingly, some acidophilic bacteria are able to reduce ferric iron also in presence of oxygen (Johnson and Bridge 2002). All of the aforementioned genera occurred in varying abundance in each of the investigated sample types. *Acidiphilium*, however, was abundant only in some of the noticeably iron-rich habitats as, e.g., in sample BFRZ2c with 14.7% and PH1 with 5.2%. Members of the genus *Acidiphilium* seem to be among the most frequently found heterotrophic acidophiles in mine-impacted environments (Johnson and McGinness 1991; Johnson et al. 2001). Its occurrence was shown to promote the growth of the widely distributed *Acidithiobacillus ferrooxidans* in several ways (Kermer et al. 2012; Liu et al. 2011). The pH prevailing in the two habitats were suitable for an initial reduction of soluble ferric iron which was shown by Coupland and Johnson (2008) to be about 2.3. The question, if *Acidiphilium* contributes to substrate regeneration for the iron oxidizers dominating the investigated communities remains to be answered. The presence of the genus *Acidithiobacillus* in high abundance suggests the participation of the species *Acidithiobacillus ferrooxidans* in iron reduction within the community. The ability of this species to reduce ferric iron to the ferrous state is well investigated (Brock and Gustafson 1976). Unfortunately, sequence analysis of the V3/V4 region is largely not sufficient for a reliable taxonomic resolution on species level.

The toxic effects that excretion of various metabolites from co-occurring microorganisms can induce in autotrophic microorganisms have been well investigated for some time (Kelly 1971; Touvinen and Kelly 1973). The removal of organic, potentially toxic substances like exudates or lysis products by heterotrophic microorganisms may promote growth of autotrophic iron oxidizers (Bacelar-Nicolau and Johnson 1999). This effect has been studied in detail for growth of *Acidithiobacillus ferrooxidans* under the influence of *Acidiphilium acidophilum* (Marchand and Silverstein 2002). The co-occurrence of *Acidiphilium* and *Ferrovum* was found in a number of the investigated habitats of RZM. A growth promoting effect through the removal of organic compounds by the heterotrophic partner was proposed in the interplay of these two taxa as well (Ullrich et al. 2015).

Interestingly, the CPla-3 termite group, only affiliated on family level was found in 17 of the investigated habitats up to a relative abundance of 3.8% (snottite BFRZ5c) and 7.1% (AMD-well S1). The CPla-3 termite group belongs to the phylum *Planctomycetota* and has been correlated with moderate and extremely acidic metal-rich environments (Ettamimi et al. 2019; Gavrilov et al. 2019). However, there are only few reports on the occurrence of the CPla-3 termite group in mine-impacted microbial communities.

With the rapid progress in tracing yet uncultivable bacteria by extensive sequencing projects a new branch in the tree of life became unveiled; the highly diverse clade Candidate Phyla Radiation (CPR) subdivides the domain bacteria (Jiao et al. 2021). The CRP comprises over 70 phyla including the superphyla Parcubacteria and Microgenomates (Danczak et al. 2017). Bacteria of the group are characterized by small cell size and genomes lacking numerous key biosynthetic pathways. This fact led to the assumption that most of these bacteria grow in association with other microorganisms adopting a mutualistic lifestyle (Castelle et al. 2018). In all of the investigated habitats of RZM phyla belonging to the monophyletic CPR were found. Sequences of the candidate phyla Eremiobacterota (formerly WPS-2) and Dormibacteraeota (formerly AD3) were detected in comparably high abundance. For instance, snottite samples BFRZ4d contained 14.1% *Ca.* Eremiobacterota and BFRZ5b 8.9% *Ca.* Dormibacteraeota, respectively. The yet uncultured *Ca.* Eremiobacterota is known to occur in bare soil environments but also in acidic and neutral mine waters (Sheremet et al. 2020; Brantner et al. 2014; Pereira et al. 2014). It was shown by metagenomics analysis that members of *Ca.* Eremiobacterota are well adapted to survive under extreme conditions in a diverse range of environments, including acidic, oligotrophic, and metal-rich habitats (Ji et al. 2021). *Ca.* Dormibacteraeota, however, seem to be less common in mine-impacted habitats (Mesa et al 2017). In both, the biofilm and the AMD samples sequences representing *Ca.* Saccharibacteria (formerly TM7) were found up to a relative abundance of 4.8% (BFRZ1b). Representatives of *Ca.* Saccharibacteria were previously reported to occur occasionally in AMD environments (Méndez-García et al. 2015). In all the described environments, *Ca.* Saccharibacteria only thrived as minor members in the microbial communities. A symbiotic lifestyle is suggested as genome analysis revealed i.a., incomplete metabolic pathways and traits of fermentative metabolism (Lemos et al. 2019).

The characterization of microbial communities thriving in habitats essentially influenced by acid mine drainage in the RZM could also become of interest for application in bioleaching. Over the past decade the number of newly founded or reopened mines in the Ore Mountains has continuously increased and different strategies for the recovery of critical raw materials are applied (Mischo and Cramer 2020). For some types of the mined ores bioleaching with selected sulfur- and iron-oxidizing bacteria from the indigenous communities could be an option for ore processing. A deeper insight into succession of bioleaching communities in mine-impacted environments and the identification of key players in the communities are prerequisites for the potential application of autochthonous microorganisms in bioleaching.

## Conclusion

A first meta-taxonomic study of prokaryotic communities from the Reiche Zeche Mine revealed with an amount of 25 phyla a surprisingly high diversity of bacteria at strongly mine-impacted locations. Although archaea were present at only few sampling sites the detection of the class *Thermoplasmata* underlines the importance the group is likely to have in mine-impacted habitats. Autotrophic iron-oxidizing bacteria *Leptospirillum* and *Acidithiobacillus* were the core taxa in the AMD-pool habitats, whereas *Ferrovum* was the dominating taxon in some of the biofilms. A great variety of other taxa involved in iron and sulfur cycling was present in most habitats. Ferric iron reducers were omnipresent but occurred at most locations only in low abundances. An unexpectedly high diversity of “microbial dark matter”, bacteria belonging to the CPR, was found. These results may help to expand our knowledge on the co-occurrence of acidophiles and members of the CPR concerning symbiotic and syntrophic relations. The obtained results on community compositions provide baseline data for operating the in situ bioleaching plant installed in the RZM. Regarding the high concentrations of some metal(loids) at many locations it seems likely to find highly resistant iron-oxidizing bacteria that could be of interest for future biomining applications on site.

## Data Availability

Sample information and corresponding 16S rRNA metagenomics data of the 25 datasets were deposited in the Sequence Read Archive (SRA) of NCBI as BioProject "Survey of microbial communities in the Reiche Zeche Mine” under accession number PRJNA706918 (BioSamples SAMN18208673 to SAMN18208697). Sequences of the V3/V4 region of the 16S rRNA gene were deposited to GenBank under the accession number SRX10272944 to SRX10272968. The metadata are accessible through the corresponding BioSample: from SAMN18208673 to SAMN18208677 for AMD pools and from SAMN18208678 to SAMN18208697 for biofilm samples, respectively.
